# Short-Term Changes, Individual Variability, and Associations with Baseline Physical Performance in Young Male Soccer Players During the Pre-Season

**DOI:** 10.3390/sports14080344

**Published:** 2026-08-10

**Authors:** Artur Avelino Birk Preissler, Filipe Manuel Clemente, Ana Filipa Silva, Rui Sousa Mendes, Pedro Schons

**Affiliations:** 1 Faculdade SOGIPA, Porto Alegre 90550-003, RS, Brazil; pedroschons@hotmail.com; 2 School of Physical Education, Physiotherapy and Dance, Federal University of Rio Grande do Sul, Porto Alegre 90690-200, RS, Brazil; 3 Applied Research Institute (i2A), Polytechnic University of Coimbra, 3045-093 Coimbra, Portugalanafilsilva@gmail.com (A.F.S.); 4 The Sport Physical Activity and Health Research & Innovation Center, 3045-093 Coimbra, Portugal; rmendes@esec.pt; 5 Coimbra Education School, Polytechnic Institute of Coimbra, Rua Dom João III-Solum, 3030-329 Coimbra, Portugal

**Keywords:** youth soccer, physical performance, pre-season, individual variability, change of direction

## Abstract

Monitoring physical performance during the pre-season may help coaches and practitioners interpret short-term changes in young soccer players, although group-level analyses may mask individual variability. This observational longitudinal study analyzed short-term changes, individual variability, and associations between baseline performance and change magnitude in 50 under-17 male outfield soccer players during a 47-day pre-season. Physical performance was assessed before and after the pre-season using the squat jump (SJ), countermovement jump (CMJ), 20 m linear sprint (Sprint-20m), 20 m change-of-direction test (COD-20m), and Yo-Yo Intermittent Recovery Test Level 1 (Yo-Yo IRL1). Significant improvements were observed in SJ (+1.43 cm, *p* < 0.001), CMJ (+1.37 cm, *p* < 0.001), and COD-20m (+0.31 km·h^−1^, *p* < 0.001), whereas no statistically significant changes were detected in Sprint-20m (−0.14 km·h^−1^, *p* = 0.121) or Yo-Yo IRL1 (−26.20 m, *p* = 0.721). Individual changes were heterogeneous, with 68.0% improving in SJ and COD-20m, 64.0% in CMJ, 40.0% in Sprint-20m, and 54.0% in Yo-Yo IRL1. Exploratory inverse associations were observed between baseline performance and change scores for SJ (ρ = −0.35, *p* = 0.014), CMJ (ρ = −0.40, *p* = 0.004), and COD-20m (ρ = −0.30, *p* = 0.033), whereas no statistically significant associations were detected for Sprint-20m or Yo-Yo IRL1. These findings support integrating group-level changes, individual variability, and baseline-performance context when interpreting short-term physical performance monitoring in youth soccer.

## 1. Introduction

Soccer performance depends on the capacity to repeat high-intensity actions within prolonged intermittent match play, including accelerations, sprints, and rapid changes in direction [1,2]. Because youth academies are expected to develop players toward increasing physical and match-play demands, routine monitoring of training load and physical performance has become central to applied sport-science practice in adolescent soccer [3]. The pre-season is a particularly informative monitoring window because training volume and conditioning emphasis are concentrated before competitive schedules, creating a plausible period for measurable short-term adaptations [4,5].

In under-17 players, physical performance monitoring commonly targets lower-limb power, linear sprint speed, change-of-direction performance, and intermittent running capacity because these domains reflect complementary neuromuscular and aerobic-anaerobic demands of soccer [1,6,7]. Squat jump and countermovement jump tests are frequently used as field measures of lower-limb explosive capacity, and their reliability and construct validity have been described in sport-science testing contexts [8]. Linear sprint and change-of-direction tests capture related but not interchangeable qualities, because athletes who sprint quickly in a straight line may not be equally efficient when decelerating, reaccelerating, and changing direction [9]. The Yo-Yo Intermittent Recovery test is relevant in this setting because it evaluates the ability to repeatedly perform intense exercise and has been widely applied in soccer populations [7,10].

A systematic review of longitudinal studies in male youth soccer reported that motor performance generally improves across adolescence, but that trajectories are shaped by biological maturation, body morphology, and training stimuli [11]. Maturation is especially important in academy contexts because maturity-related differences in anthropometry, neuromuscular performance, training tolerance, and injury risk can be substantial around peak height velocity [12,13]. Recent systematic reviews and meta-analyses indicate that supplementary plyometric, strength, and combined training can improve jump, sprint, and change-of-direction outcomes in young soccer players, although the magnitude of benefit depends on the training modality and performance domain [14,15,16]. Longitudinal and pre-season studies also suggest that performance change is not uniform across tests, with intermittent running, repeated-sprint, power, and speed-related outcomes showing different developmental and training-associated patterns [4,17]. In the context of the present study, maturation was therefore considered a contextual factor that could contribute to the differences in physical performance and the changes observed among the players.

Despite the practical value of monitoring, reliance on group mean changes can obscure practically relevant differences in the direction and magnitude of individual responses [18,19]. This issue is particularly salient in youth soccer because players within the same chronological age category can differ substantially in maturity status, body size, positional demands, training history, and baseline physical capacity [6,12,13]. Evidence from male youth soccer plyometric training shows that individual change patterns can diverge even when players complete the same intervention, supporting the need to describe variability rather than infer uniform adaptation [18]. Baseline–change associations may provide additional context for interpreting observed changes, but they require caution because apparent relationships may reflect true differences, ceiling effects, regression to the mean, or measurement error [19,20], while correlations involving change scores may also be affected by mathematical coupling. Although previous studies have examined longitudinal changes or individual response patterns, the combined interpretation of group-level changes, individual change distributions, and baseline–change associations within a single applied pre-season monitoring framework remains insufficiently described in under-17 academy players.

Accordingly, the present study aimed to analyze short-term changes, individual variability, and associations between baseline performance and change magnitude in the physical performance of young male soccer players during the pre-season. Maturity offset was included as a contextual variable and examined in complementary analyses of its associations with the observed changes. We hypothesized that selected physical capacities would improve over the pre-season and that individual changes would be heterogeneous [11,18]. Associations between baseline performance and change magnitude were treated as exploratory rather than as direct evidence of differential responsiveness, given the potential influence of regression to the mean and measurement error. These associations were also interpreted cautiously because change scores are mathematically dependent on baseline values.

## 2. Materials and Methods

### 2.1. Participants

Male outfield soccer players were recruited from a competitive youth academy whose under-17 team participated in the main state-level competition for this age category. The investigation followed an observational, longitudinal, descriptive, and quantitative design during the pre-season. Convenience sampling was adopted, and inclusion in the analysed sample required availability to complete the physical-testing battery at both assessment time points. The final sample comprised 50 under-17 players: midfielders (*n* = 17), forwards (*n* = 14), centre-backs (*n* = 11), and full-backs (*n* = 8). At baseline, the players were 14.24 ± 1.00 years old and reported 7.28 ± 2.14 years of soccer practice. Their stature was 171.00 ± 8.38 cm, sitting height was 87.84 ± 4.87 cm, body mass was 60.28 ± 8.93 kg, and estimated maturity offset was 0.61 ± 0.91 years relative to peak height velocity. Five goalkeepers were not included because of the position-specific physical demands of their playing role. Players who did not have complete paired data from the pre- and post-assessments were also excluded. All procedures complied with the principles of the Declaration of Helsinki. Before participation, written informed consent was provided by the players’ legal guardians, and assent was obtained from the players. Approval was obtained before data collection from the Research Ethics Committee of the Pontifical Catholic University of Rio Grande do Sul (CEP-PUCRS) (Approval number: 7.047.156; date of approval: 2 September 2024).

### 2.2. Design and Setting

Testing was integrated into the team’s usual pre-season environment and was performed on two occasions, with 47 days between assessments. During the intervening period, the players continued their regular pre-season schedule, which involved approximately five training days per week and sessions of around two hours combining technical, tactical, and physical activities. The content and intensity of individual sessions were not prospectively documented, and neither internal nor external training load was measured. Both assessments were conducted at the same time of day after a minimum of 24 h without training. Environmental conditions were not formally recorded. As testing was part of the team’s established performance-monitoring routine, no separate familiarisation session was conducted. The sequence and administration of the tests were maintained across the two assessment occasions. Before starting the battery, players completed the dynamic mobility exercises, low-intensity running, and progressive accelerations routinely used by the team and then received verbal instructions from the evaluators.

### 2.3. Procedures

The assessment battery included body mass, stature, sitting height, estimated maturity offset relative to peak height velocity, squat jump (SJ), countermovement jump (CMJ), 20 m linear sprint (Sprint-20m), 20 m change-of-direction test (COD-20m), and the Yo-Yo Intermittent Recovery Test Level 1 (Yo-Yo IRL1). The same testing sequence was followed at both time points to reduce variation attributable to test order. For Sprint-20m and COD-20m, the shortest completion time was transformed into average velocity (km·h^−1^) according to velocity = distance/time × 3.6. This conversion placed all outcomes in the same direction, with higher values indicating better performance. Two trials were completed for SJ, CMJ, Sprint-20m, and COD-20m. This number of attempts was selected to allow a valid maximal effort while limiting fatigue and interference with the team’s regular training routine. The best valid result from each test was included in the analyses.

#### 2.3.1. Anthropometric Assessment

Body mass, standing stature, and sitting height were recorded. Body mass was obtained with a digital scale with a resolution of 0.1 kg (G-TECH, Accumed Produtos Médico Hospitalares Ltd., Duque de Caxias, Brazil). Players were assessed barefoot and wearing light clothing. Standing stature was measured with a non-extensible tape secured vertically to a flat wall and providing a resolution of 1 mm. During this measurement, players remained barefoot and upright, with their heels together and their head positioned in the Frankfurt plane. The same tape was used to determine sitting height. Players sat on a standardized bench with the trunk upright and the head maintained in the Frankfurt plane. When required, bench height was deducted to calculate the distance from the sitting surface to the vertex of the head. Chronological age was derived from the date of birth and the date of the baseline assessment, whereas soccer-practice experience was self-reported in years. Maturity offset relative to peak height velocity was estimated using the anthropometric prediction equation [21]. In accordance with the subsequent clarification [22], the body mass-to-stature ratio was multiplied by 100 when the equation was applied. The equation incorporates chronological age, body mass, stature, sitting height, and leg length, with leg length obtained by subtracting sitting height from stature. Maturity offset was reported in years relative to peak height velocity. Negative estimates denoted a period before peak height velocity, whereas positive estimates denoted a period after peak height velocity.

#### 2.3.2. Vertical Jump Test

Performance in the squat jump and countermovement jump was used to assess lower-limb power. Both tests were performed on a contact platform (Jump System, Cefise, Nova Odessa, Brazil) connected to a portable computer. Arm-swing contribution was restricted by instructing players to keep their hands on their hips during both jumps. For the SJ, players assumed a stationary semi-squat position with approximately 90° of knee flexion before jumping as high as possible without a preceding countermovement. For the CMJ, players began upright and, after a verbal command, rapidly descended to approximately 90° of knee flexion before immediately performing a maximal extension of the lower limbs [23,24]. Two trials of each jump were completed, separated by 30 s, and the highest valid result was selected. Jump height was reported in centimeters and calculated by the system from flight time according to *h* = *g* × *t*^2^/8, where *h* is jump height, *g* is gravitational acceleration, and *t* is flight time. Evaluators used verbal instructions and visual inspection during each attempt to standardize the approximate 90° knee-flexion position. At baseline, the intraclass correlation coefficient and Cronbach’s α were 0.861 and 0.935 for SJ and 0.815 and 0.897 for CMJ, respectively.

#### 2.3.3. Linear Sprint Test

Linear sprint performance was evaluated over 20 m using the Sprint-20m test. Times were recorded on the field with an electronic photocell system (CEFISE, São Paulo, Brazil) with a precision of 1 ms. The photocells were installed 100 cm above the ground. At the start, players positioned their front foot 0.30 m behind the first gate and maintained a slight forward inclination of the trunk. On an auditory signal, they accelerated and sprinted maximally through the 20 m course. Timing gates were located at 0 and 20 m, and a cone positioned 5 m beyond the final gate encouraged players to continue running through the finish line [25,26]. Two trials were performed, with 3 min of passive recovery between them. The shortest recorded time was used for analysis and was converted into average velocity (km·h^−1^) over the 20 m distance. At baseline, the intraclass correlation coefficient and Cronbach’s α for Sprint-20m were 0.828 and 0.904, respectively.

#### 2.3.4. Change of Direction Test

Change-of-direction performance was evaluated with the COD-20m test. The 20 m course was arranged as a zig-zag comprising four successive 5 m sections. Each directional transition was indicated by a cone; adjacent sections formed angles of approximately 100°, and players ran around the outside of every cone. They were instructed to complete the course as quickly as possible. Performance was timed with an electronic photocell system (CEFISE, São Paulo, Brazil), with the gates positioned 100 cm above the ground at the beginning and end of the course. Players started with the front foot 0.30 m behind the first gate. A cone located 5 m after the finishing gate was used to discourage deceleration before the line [25,26]. Each player completed two trials separated by 3 min of passive recovery. The shortest time was retained and converted into average velocity (km·h^−1^) for the 20 m course. At baseline, the intraclass correlation coefficient and Cronbach’s α for COD-20m were 0.856 and 0.925, respectively.

#### 2.3.5. Yo-Yo Intermittent Recovery Test Level 1

The Yo-Yo Intermittent Recovery Test Level 1 (Yo-Yo IRL1) was used to evaluate intermittent-running performance. Players completed successive 2 × 20 m shuttle runs at speeds prescribed by audio signals and increased progressively throughout the test. Running bouts were interspersed with 10 s of active recovery within a 2 × 5 m area. Cones delimited the 20 m running lanes and the recovery zone. Players were instructed to follow the audio-prescribed pace for as long as they were able. Testing ended when a player stopped voluntarily because of exhaustion or failed on two occasions to reach the required line at the appropriate audio signal. The total distance completed was expressed in meters and served as the test outcome [10].

### 2.4. Statistical Analysis

Data were summarized as mean ± standard deviation. For each physical test, individual change (Δ) was obtained by subtracting the pre-assessment value from the post-assessment value. The distribution of the change scores was evaluated with the Shapiro–Wilk test. Because Δ values for SJ and COD-20m did not meet the normality assumption, differences between assessments were examined with the Wilcoxon signed-rank test. Paired *t*-tests were used for CMJ, Sprint-20m, and Yo-Yo IRL1. The effect-size measure was selected according to the inferential test: Cohen’s d was used with paired *t*-tests, and rank-biserial correlation was used with Wilcoxon signed-rank tests. Individual change distributions were characterized by the median Δ, interquartile range (Q1–Q3), minimum and maximum, and the number and percentage of players with positive, zero, or negative changes. These observations were labelled improved (Δ > 0), unchanged (Δ = 0), and worsened (Δ < 0), respectively. The labels described only the direction of the measured change and were not used to identify true responders or non-responders. Spearman’s rank correlation coefficient was used to examine associations between baseline performance and subsequent change. The primary correlation analyses paired the baseline value of each physical test with its corresponding Δ. Estimated maturity offset was considered a contextual variable rather than a covariate in the primary pre–post analyses; its associations with the change scores were therefore examined separately using Spearman’s rank correlation coefficient. Absolute Spearman’s ρ values were interpreted according to the following criteria: trivial (<0.10), small (0.10–0.29), moderate (0.30–0.49), large (0.50–0.69), very large (0.70–0.89), and nearly perfect (≥0.90) [19]. Baseline–change correlations were considered exploratory because no adjustment for multiple comparisons was applied and because mathematical coupling and regression to the mean could influence these associations. Within-session reliability at baseline was assessed using a two-way absolute-agreement intraclass correlation coefficient based on single measurements and Cronbach’s α for SJ, CMJ, Sprint-20m, and COD-20m. Statistical significance was established at α = 0.05. Analyses were conducted in Jamovi, version 2.6.26.

## 3. Results

The results of the pre- and post-assessments, mean changes (Δ), statistical tests, *p*-values, and effect sizes for each physical performance test are presented in Table 1. Because Sprint-20m and COD-20m performances were expressed as average velocity (km·h^−1^), all variables were organized so that higher values represented better performance. Therefore, positive Δ values indicated improvement in all physical tests. Statistically significant improvements were detected for SJ, CMJ, and COD-20m. No statistically significant changes were detected for Sprint-20m or Yo-Yo IRL1. At the group level, lower-limb power and change-of-direction performance improved, whereas changes in linear sprint and intermittent-running performance were not statistically significant.

**Figure 1:**
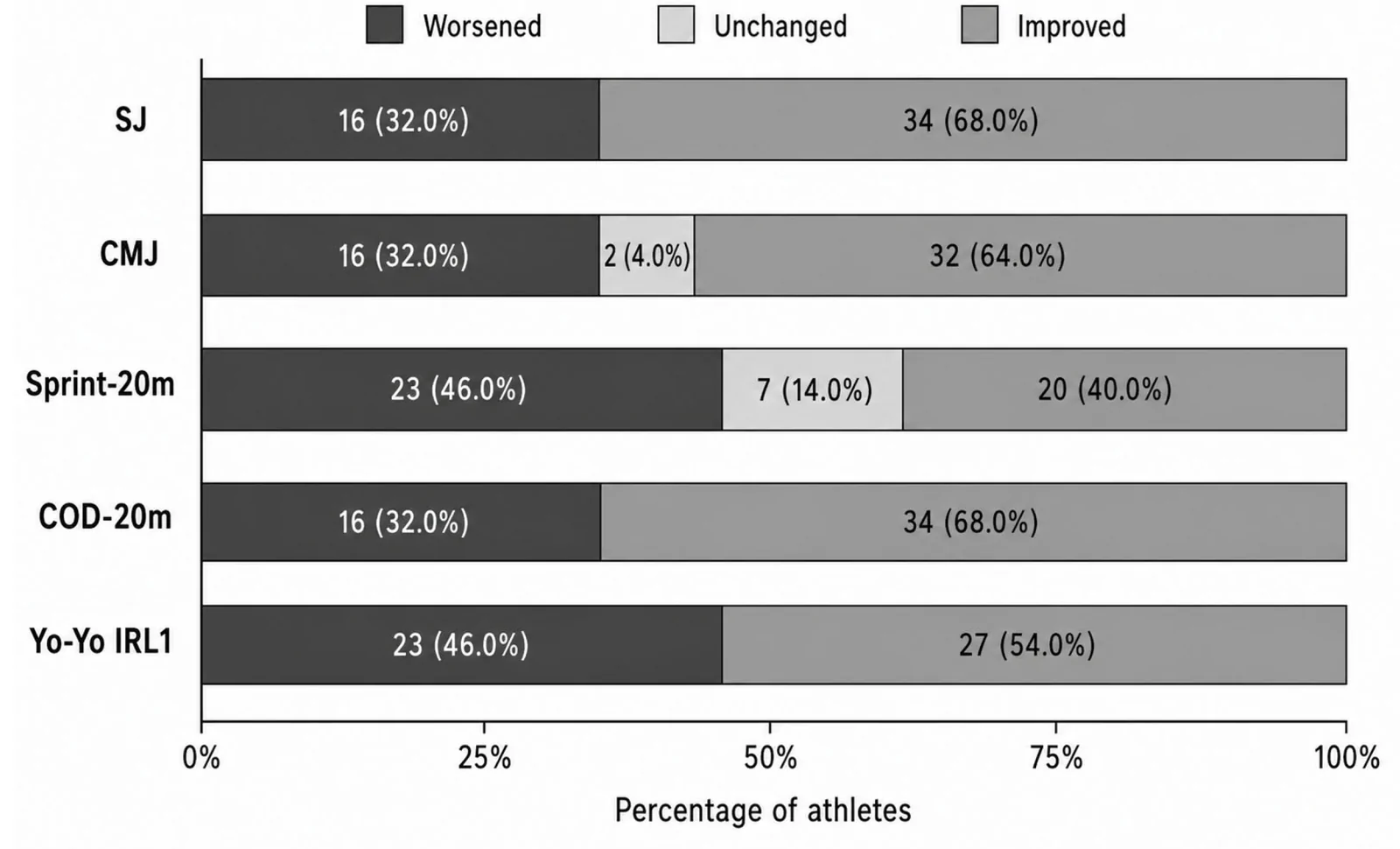
Proportion of athletes who worsened, remained unchanged, or improved in each physical performance test. Bars represent the percentage of athletes classified as worsened (Δ < 0), unchanged (Δ = 0), or improved (Δ > 0). Because all variables were expressed so that higher values represented better performance, positive Δ values indicate improvement. SJ = Squat Jump; CMJ = Countermovement Jump; Sprint-20m = 20 m linear sprint; COD-20m = 20 m change-of-direction test; Yo-Yo IRL1 = Yo-Yo Intermittent Recovery Test Level 1.

Table 2 presents the individual distribution of change scores (Δ) for each physical performance test. This analysis was performed to complement the interpretation of the group mean changes by describing the individual variability observed during the study period. Considerable individual variability was observed across all tests. Even in SJ, CMJ, and COD-20m, which showed significant group-level improvements, some athletes exhibited performance declines. In Yo-Yo IRL1, although no significant group-level change was detected, 27 athletes improved whereas 23 showed lower post-assessment performance. Overall, 29 athletes improved in at least three of the five physical tests, whereas 21 improved in two or fewer variables. Figure 1 complements Table 2 by illustrating the proportion of athletes who improved, remained unchanged, or worsened in each physical performance test, highlighting the heterogeneity of the individual responses.

Table 3 presents the associations between baseline performance and the magnitude of the observed changes. Negative coefficients indicated inverse associations between baseline values and the corresponding change scores. Moderate negative correlations were observed for SJ, CMJ, and COD-20m. These exploratory associations should be interpreted cautiously because baseline values are mathematically related to change scores and no adjustment for multiple comparisons was applied. No statistically significant associations were detected for Sprint-20m or Yo-Yo IRL1. As a complementary analysis, baseline-estimated maturity offset was not significantly associated with the magnitude of change in any physical performance test. Correlations between baseline maturity offset and change scores were trivial to small for SJ (ρ = −0.08; *p* = 0.599), CMJ (ρ = −0.10; *p* = 0.505), Sprint-20m (ρ = −0.09; *p* = 0.556), COD-20m (ρ = −0.17; *p* = 0.230), and Yo-Yo IRL1 (ρ = 0.07; *p* = 0.644).

## 4. Discussion

The present study examined short-term changes, individual variability, and baseline–change associations in physical performance in under-17 male outfield soccer players during a 47-day pre-season period. The main finding was a domain-specific pattern: lower-limb power and change-of-direction performance improved at the group level, whereas no statistically significant changes were detected in 20-m linear sprint velocity or Yo-Yo IRL1 distance. Individual changes were heterogeneous across all tests, and exploratory inverse associations were observed between baseline performance and change scores for SJ, CMJ, and COD-20m, whereas no statistically significant associations were detected for Sprint-20m or Yo-Yo IRL1. Collectively, these findings partially support the study hypothesis and indicate that short-term pre-season monitoring should not be interpreted only through mean group changes. From a practical perspective, the relevance of these changes should be considered alongside their absolute magnitude, reported effect sizes, and individual distributions, because statistically significant group-level improvements were not observed in every player.

The increases observed in SJ and CMJ indicate that lower-limb explosive performance changed positively over the monitored pre-season period. This finding agrees with longitudinal evidence showing that motor performance in male youth soccer generally improves across development, although trajectories are influenced by maturation, body composition, and training exposure [11]. It also accords with academy monitoring data showing that sprint and jump qualities vary across seasonal periods and age groups [27]. Evidence in high-level male youth soccer indicates that strength, plyometric, and combined training can improve squat jump and countermovement jump performance, with particularly consistent effects for power-related outcomes [15]. A systematic review of adolescent soccer players further reported moderate effects of plyometric training on jumping and change-of-direction performance, but only small effects on sprinting, suggesting that neuromuscular and multidirectional outcomes may be more readily changed over short training windows than linear speed [14]. Mixed technical, tactical, and physical exposure is commonly present during preparatory periods in youth soccer [28]; however, because training content and load were not quantified, the observed changes cannot be attributed to any specific component of the pre-season program.

The improvement in COD-20m extends the neuromuscular findings by showing that the pre-season period was also accompanied by better performance in a multidirectional field test. This result is coherent with evidence that change-of-direction performance in young soccer players is associated with, but not reducible to, sprint and jump abilities [29,30]. Change-of-direction tasks require braking, body reorientation, reacceleration, and segmental control, which may explain why COD performance can change differently from linear sprint performance [29,30]. Meta-analyses also show that plyometric and complex training can improve COD performance in soccer players, supporting the plausibility of COD improvement during periods that include substantial neuromuscular and multidirectional content [14,31]. However, because COD was expressed as average velocity in a specific 20-m zig-zag test, the present finding should be interpreted as test-specific rather than as a comprehensive indicator of reactive agility or match-play directional skill [30].

The statistical pattern differed across physical domains over the 47-day period, because statistically significant changes were detected for the jump and COD outcomes but not for Sprint-20m or Yo-Yo IRL1. This divergence is consistent with meta-analytic evidence showing that plyometric training effects in adolescent soccer players are larger for jump and COD outcomes than for sprint performance [14]. It is also partly consistent with pre-season data in elite players aged 17–19 years, in which power improved after eight weeks but U-17 sprint performance and Yo-Yo IR1 distance did not show significant improvement [32]. Linear sprint performance may require sufficient exposure to maximal or near-maximal velocity running and technical sprint stimuli, which cannot be inferred from a generic description of mixed pre-season training [15,28]. Yo-Yo IRL1 performance reflects the ability to repeatedly perform intense intermittent exercise with substantial aerobic loading and recovery demands [7,10,33]. Because training content and load were not quantified, the absence of a statistically significant group-level change cannot be attributed to insufficient, non-specific, or variable training exposure. These null findings should not be interpreted as definitive evidence of absent physiological adaptation, but rather as evidence that mean change in these field-test outputs was not statistically detectable in this sample and context.

The individual distributions were a central finding because group-level improvements in SJ, CMJ, and COD-20m coexisted with performance declines in a sizeable subset of players. This observation agrees with evidence from youth soccer plyometrics showing substantial interindividual variability after the same training exposure [18]. The negative baseline–change correlations observed for SJ, CMJ, and COD-20m should not be interpreted as direct evidence that initially lower-performing players were more responsive, as these associations may partly reflect regression to the mean and measurement error [34,35]. Because each change score was calculated using its corresponding baseline value, mathematical coupling may also have contributed to the inverse associations. Exercise-training methodology papers emphasize that claims about individual response require designs or analyses that separate true response heterogeneity from random within-subject variation [34,35]. A systematic review of individual-response methods further concluded that studies should account for measurement error and the smallest worthwhile or clinically meaningful change when classifying responders and non-responders [36]. Accordingly, the improved, unchanged, and worsened categories in this study are best interpreted as descriptive directions of observed change rather than evidence of true responder status. The lack of statistically significant associations between maturity offset and change scores should be interpreted cautiously, because maturation influences physical performance and training tolerance in youth soccer and maturity-offset predictions can be imprecise at the individual level [12,37].

This study has limitations. The convenience sample limits external validity, and differences in age, maturity status, playing position, and training history may have contributed to the observed heterogeneity. Most importantly, the observational pre–post design and absence of a control group preclude causal attribution of the observed changes to pre-season training, particularly because growth- and maturation-related development may also have influenced physical performance in this adolescent cohort [12]. Training content, intensity, and internal and external load were not quantified, limiting the interpretation of how training exposure was related to the observed changes [28] and preventing these changes from being linked to specific training stimuli. Future studies should use multicenter samples, repeated assessments across the season, controlled or well-characterized training exposure, and objective internal and external load measures. Studies should also incorporate maturity, wellness, injury, and training and match exposure because these biological and contextual factors are relevant to the interpretation and management of physical performance in youth soccer [3,12]. Technical–tactical variables should also be considered to provide a broader contextual interpretation of physical monitoring. Moreover, the exploratory baseline–change correlations were not adjusted for multiple comparisons and may have been influenced by mathematical coupling and regression to the mean. Although within-session reliability was estimated, the descriptive classification of individual changes did not incorporate a longitudinal measurement-error threshold [19,20] or a smallest worthwhile change [36].

From an applied perspective, the findings support a cautious and individualized interpretation of pre-season monitoring. Coaches and practitioners should view improvements in lower-limb power and COD as useful but incomplete indicators of short-term physical development because no statistically significant changes were detected in sprint or intermittent-running performance over the same period. This domain-specific pattern suggests that pre-season monitoring batteries should retain complementary tests rather than relying on a single measure of physical readiness. Baseline scores may provide useful context for identifying players who warrant closer monitoring, but baseline–change correlations should not be used alone to label athletes as responders because error and regression to the mean can distort individual change interpretation [34,35]. A multidisciplinary approach that considers maturation alongside other biological and contextual factors is preferable when interpreting physical monitoring data and adapting training priorities for adolescent players.

## 5. Conclusions

Young male soccer players showed short-term group-level improvements in lower-limb power and change-of-direction performance during the pre-season, whereas no statistically significant changes were detected in linear sprint or intermittent running performance. Individual changes were heterogeneous across all tests, indicating that group means did not fully represent player-level patterns. Exploratory inverse associations were observed between baseline performance and change scores for SJ, CMJ, and COD-20m; however, these findings should be interpreted cautiously in light of the study limitations. These findings are specific to the present sample, academy context, and 47-day monitoring period and support the integration of group-level changes and individual variability when interpreting pre-season physical performance monitoring.

## Figures and Tables

**Table 1:** Changes in physical performance between pre- and post-assessment.

Variable	Pre	Post	Mean Δ	*p*-Value	Effect Size
SJ (cm)	32.34 ± 5.02	33.77 ± 4.64	+1.43	<0.001 *	0.55
CMJ (cm)	33.82 ± 5.39	35.19 ± 5.04	+1.37	<0.001 *	0.50
Sprint-20m (km·h^−1^)	23.30 ± 1.24	23.16 ± 1.28	−0.14	0.121	−0.22
COD-20m (km·h^−1^)	13.43 ± 0.60	13.75 ± 0.67	+0.31	<0.001 *	0.57
Yo-Yo IRL1 (m)	1749.80 ± 515.10	1723.60 ± 600.46	−26.20	0.721	−0.05

Note: Values are presented as mean ± standard deviation. Δ = post-assessment minus pre-assessment. SJ = Squat Jump; CMJ = Countermovement Jump; Sprint-20m = 20 m linear sprint; COD-20m = 20 m change-of-direction test; Yo-Yo IRL1 = Yo-Yo Intermittent Recovery Test Level 1; * Statistically significant pre- to post-assessment difference (*p* < 0.05). Wilcoxon signed-rank tests were used for SJ and COD-20m, whereas paired *t*-tests were used for CMJ, Sprint-20m, and Yo-Yo IRL1. Effect sizes correspond to the rank-biserial correlation for Wilcoxon tests and Cohen’s d for paired *t*-tests, and were reported in the same direction as Δ.

**Table 2:** Individual distribution of observed changes in physical performance.

Variable	Median Δ	Q1–Q3	Min–Max	Worsened	Unchanged	Improved
SJ (cm)	+1.70	−0.58 to 3.38	−7.50 to 12.20	16 (32.0%)	0 (0.0%)	34 (68.0%)
CMJ (cm)	+1.50	−0.73 to 3.15	−3.80 to 7.60	16 (32.0%)	2 (4.0%)	32 (64.0%)
Sprint-20m (km·h^−1^)	0.00	−0.69 to 0.30	−1.40 to 1.35	23 (46.0%)	7 (14.0%)	20 (40.0%)
COD-20m (km·h^−1^)	+0.22	−0.11 to 0.59	−0.67 to 2.53	16 (32.0%)	0 (0.0%)	34 (68.0%)
Yo-Yo IRL1 (m)	+90	−425 to 255	−1240 to 860	23 (46.0%)	0 (0.0%)	27 (54.0%)

Note: Δ = post-assessment minus pre-assessment. Q1–Q3 = interquartile range. Min–Max = minimum and maximum individual change scores. SJ = Squat Jump; CMJ = Countermovement Jump; Sprint-20m = 20 m linear sprint; COD-20m = 20 m change-of-direction test; Yo-Yo IRL1 = Yo-Yo Intermittent Recovery Test Level 1. Worsened = Δ < 0; Unchanged = Δ = 0; Improved = Δ > 0. Because all variables were expressed so that higher values represented better performance, positive Δ values indicate improvement. This classification describes only the direction of the observed changes and should not be interpreted as the identification of true responders or non-responders.

**Table 3:** Associations between baseline performance and magnitude of observed changes.

Relationship	Spearman’s ρ	*p*-Value
SJ baseline × ΔSJ	−0.35	0.014 *
CMJ baseline × ΔCMJ	−0.40	0.004 *
Sprint-20m baseline × ΔSprint-20m	−0.15	0.306
COD-20m baseline × ΔCOD-20m	−0.30	0.033 *
Yo-Yo IRL1 baseline × ΔYo-Yo IRL1	−0.26	0.069

Note: ρ = Spearman’s rank correlation coefficient. Δ = post-assessment minus pre-assessment. SJ = Squat Jump; CMJ = Countermovement Jump; Sprint-20m = 20 m linear sprint; COD-20m = 20 m change-of-direction test; Yo-Yo IRL1 = Yo-Yo Intermittent Recovery Test Level 1; * Statistically significant association (*p* < 0.05). Negative coefficients indicate inverse associations between baseline values and the corresponding change scores.

## Data Availability

The data presented in this study are available upon reasonable request from the corresponding author. The data are not publicly available due to privacy and ethical restrictions related to the potential identification of youth soccer players.
